# Comparative Genomics of *Lactiplantibacillus plantarum*: Insights Into Probiotic Markers in Strains Isolated From the Human Gastrointestinal Tract and Fermented Foods

**DOI:** 10.3389/fmicb.2022.854266

**Published:** 2022-05-18

**Authors:** Natalia Garcia-Gonzalez, Francesca Bottacini, Douwe van Sinderen, Cormac G. M. Gahan, Aldo Corsetti

**Affiliations:** ^1^Faculty of Bioscience and Technology for Food, Agriculture and Environment, University of Teramo, Teramo, Italy; ^2^School of Microbiology, University College Cork, Cork, Ireland; ^3^Synbiotec S.r.l., Spin-off of University of Camerino, Camerino, Italy; ^4^APC Microbiome Ireland, University College Cork, Cork, Ireland; ^5^Biological Sciences, Munster Technological University, Cork, Ireland; ^6^School of Pharmacy, University College Cork, Cork, Ireland

**Keywords:** genome sequencing, lactic acid bacteria, *Lactiplantibacillus plantarum*, probiotic, food-borne bacteria

## Abstract

*Lactiplantibacillus* (*Lpb.) plantarum* is a versatile species commonly found in a wide variety of ecological niches including dairy products and vegetables, while it may also occur as a natural inhabitant of the human gastrointestinal tract. Although *Lpb. plantarum* strains have been suggested to exert beneficial properties on their host, the precise mechanisms underlying these microbe–host interactions are still obscure. In this context, the genome-scale *in silico* analysis of putative probiotic bacteria represents a bottom–up approach to identify probiotic biomarkers, predict desirable functional properties, and identify potentially detrimental antibiotic resistance genes. In this study, we characterized the bacterial genomes of three *Lpb. plantarum* strains isolated from three distinct environments [strain IMC513 (from the human GIT), C904 (from table olives), and LT52 (from raw-milk cheese)]. A whole-genome sequencing was performed combining Illumina short reads with Oxford Nanopore long reads. The phylogenomic analyses suggested the highest relatedness between IMC513 and C904 strains which were both clade 4 strains, with LT52 positioned within clade 5 within the *Lpb. plantarum* species. The comparative genome analysis performed across several *Lpb. plantarum* representatives highlighted the genes involved in the key metabolic pathways as well as those encoding potential probiotic features in these new isolates. In particular, our strains varied significantly in genes encoding exopolysaccharide biosynthesis and in contrast to strains IMC513 and C904, the LT52 strain does not encode a Mannose-binding adhesion protein. The LT52 strain is also deficient in genes encoding complete pentose phosphate and the Embden–Meyerhof pathways. Finally, analyses using the CARD and ResFinder databases revealed that none of the strains encode known antibiotic resistance loci. Ultimately, the results provide better insights into the probiotic potential and safety of these three strains and indicate avenues for further mechanistic studies using these isolates.

## Introduction

*Lactiplantibacillus* (*Lpb*.) *plantarum* (Zheng et al., 2020) is a Gram-positive, non-motile, non-spore-forming, microaerophilic, and mesophilic bacterium that belongs to the lactic acid bacteria (LAB). It is one of the most versatile species among LAB and this is reflected in its capacity to colonize a wide number of niches such as the gastrointestinal and vaginal tracts, vegetables, dairy products, and fermented foods (Garcia-Gonzalez et al., 2021a). It is widely used in industrial fermentation since it is “Generally Recognized as Safe” (GRAS) and has Qualified Presumption of Safety (QPS) status (Guidone et al., 2014; Ricci et al., 2017). Over the last century, interest in the applications of *Lpb. plantarum* strains has been reinforced by their documented functional and health-promoting properties (Seddik et al., 2017; Behera et al., 2018). Beneficial properties attributed to *Lpb. plantarum* are diverse, varying from cholesterol-lowering activity (Kumar et al., 2011) to enhancement of the intestinal barrier and modulation of the commensal microbiota (Yang et al., 2014). In particular, the three strains evaluated in the current work have shown potential health benefits in several studies, demonstrating *in vitro* anti-inflammatory properties and the potential to survive the passage through the human gastrointestinal tract (Garcia-Gonzalez et al., 2018, 2021b; Prete et al., 2020).

According to the FEEDAP Guidance document (EFSA FEEDAP Panel, 2018) and the EFSA’s statement, an unequivocal taxonomic identification at the strain level has to be performed for all microorganisms intentionally used in the food chain^1^. Even within the same species, differences between strains may be significant and the properties assigned to one strain cannot necessarily be extrapolated to another. For this reason, data obtained from the whole-genome sequence (WGS) and WGS-based data analysis is a requirement for the characterization of bacterial and yeast strains used in the food chain. WGS can provide valuable information regarding the characterization of the potential functional traits of these strains as well as information related to virulence factors, resistance to antimicrobials, and the production of toxic metabolites.

Previous analysis of the available *Lpb. plantarum* genome sequences has revealed a high level of genomic diversity, versatility, and plasticity, which may facilitate highly successful adaptation of *Lpb. plantarum* strains to diverse niches (Siezen and van Hylckama Vlieg, 2011). This genome-driven adaptability is also due to the ability of the *Lpb. plantarum* genomes to acquire the so-called carbohydrate utilization islands, which represent gene clusters that allow growth on particular carbohydrates present in specific niches (Kleerebezem et al., 2003). Due to the increased interest regarding the impact of *Lpb. plantarum* on human health, and the necessity to identify genetic determinants associated with probiotic properties, the genome sequences of three *Lpb. plantarum* strains isolated from different sources were performed. We have selected these strains based on their abundance in foods (Licitra and Carpino, 2014; Perpetuini et al., 2020) and our initial findings that they may be immunomodulatory if consumed in adequate quantities (Garcia-Gonzalez et al., 2018, 2021b). In particular, we note that strains that are abundant in certain foods may be consumed at high levels (up to 10^8^ per gram), even though relatively little is known about how such strains may impact human health. Here, we present the draft genome sequences of one probiotic *Lpb. plantarum* strain (isolated from the human GI tract), IMC513, and two food-related strains, C9O4 and LT52, representative of isolates found at high levels in olives and raw-milk cheese, respectively. The aim of this study is to provide an overview of the genomic content of our strains with a particular emphasis on the potential to encode probiotic and immunomodulatory properties which could inform future mechanistic studies. We also wished to determine the potential for isolates to encode mobilizable antibiotic resistance loci or potential virulence factors to conform to best practices for the future safe utilization of such strains as probiotics.

## Materials and Methods

### Bacterial Strains and Growth Conditions

The food-related *Lpb. plantarum* strains used in this study were obtained from the laboratory collection at the University of Teramo (Table 1). A commercial probiotic strain, *Lpb. plantarum* IMC513, was included in the study as a human-derived reference strain (Synbiotec, Camerino, Italy). All isolates were grown in the MRS broth (Oxoid) at 37°C.

**TABLE 1:** Genome assembly data after Nanopore and Illumina sequencing.

Strain	Source	Number of reads	# contig (≥0 bp)	# contig (≥1000 bp)	# contig	Largest contig	Total length (bp)	N50*
IMC513	GIT	598,282	47	23	25	549,450	3,303,141	369,769
C9O4	Table olives	481,788	53	34	37	454,255	3,352,718	211,991
LT52	Raw-milk cheese	443,179	56	25	28	812,920	3,282,907	411,061

*N50 is defined as the length contigs (N) for which 50% of all bases in the assembly are in a sequence of length L < N.

*GIT, Gastrointestinal tract.*

### Genome Sequencing and Annotation

Genomic DNA isolation and sequencing were performed by Microbes NG (University of Birmingham, United Kingdom). The DNA of the three *Lpb. plantarum* strains was isolated from pure cultures and whole-genome sequencing was performed using a combined the Illumina short reads and Oxford Nanopore long reads approach (Table 1).

Method SGS (Illumina): Plated cultures of each isolate were inoculated into a cryo-preservative (Microbank™, Pro-Lab Diagnostics UK, United Kingdom). About 10–20 μl of the suspension was lysed with 120 μl of TE buffer containing lysozyme (final concentration 0.1 mg/ml) and RNase A (ITW Reagents, Barcelona, Spain) (final concentration 0.1 mg/ml), incubated for 25 min at 37°C. Proteinase K (VWR Chemicals, OH, United States; final concentration 0.1 mg/ml) and SDS (Sigma–Aldrich, MO, United States; final concentration 0.5% v/v) were added and incubated for 5 min at 65°C. Genomic DNA was purified using an equal volume of SPRI beads and resuspended in EB buffer (Qiagen, Germany). The DNA was quantified with the Quant-iT dsDNA HS kit (ThermoFisher Scientific, Waltham, MA, United States) assay in an Eppendorf AF2200 plate reader (Eppendorf UK Ltd., United Kingdom). Genomic DNA libraries were prepared using the Nextera XT Library Prep Kit (Illumina, San Diego, CA, United States) following the manufacturer’s protocol with the following modifications: 2 ng of DNA was used as input, and the PCR elongation time was increased to 1 min from 30 s. The DNA quantification and library preparation were carried out on a Hamilton Microlab STAR automated liquid handling system (Hamilton Bonaduz AG, Switzerland). Pooled libraries were quantified using the Kapa Biosystems Library Quantification Kit for Illumina on a Roche light cycler 96 qPCR machine. The libraries were sequenced with the Illumina HiSeq using a 250 bp paired-end protocol.

Method ONT sequencing for EGS: Broth cultures of each isolate were pelleted out and the pellet was resuspended in the cryo-preservative of a Microbank™ (Pro-Lab Diagnostics UK, United Kingdom) tube and stored in the tube. Approximately 2 × 10^9^ cells were used for high molecular weight DNA extraction using Nanobind CCB Big DNA Kit (Circulomics, MD, United States). The DNA was quantified with the Qubit dsDNA HS assay in a Qubit 3.0 (Invitrogen) Eppendorf UK Ltd, United Kingdom). Long read genomic DNA libraries were prepared with Oxford Nanopore SQK-RBK004 kit and/or SQK-LSK109 kit with Native Barcoding EXP-NBD104/114 (ONT, United Kingdom) using 400–500 ng of HMW DNA. About 12–24 barcoded samples were pooled together into a single sequencing library and loaded in a FLO-MIN106 (R.9.4 or R.9.4.1) flow cell in a GridION (ONT, United Kingdom).

The Illumina reads were adapter trimmed using Trimmomatic 0.30 with a sliding window quality cutoff of Q15 (Bolger et al., 2014). Raw sequence data were filtered and assembled using Unicycler v0.4.8 (Wick et al., 2017). Genome annotation was performed *via* the Prokka annotation server with default parameters (Seemann, 2014).

### Bioinformatic Analysis

Comparative genome analysis was performed on three *Lpb. plantarum* strains sequenced within this study and 39 fully sequenced *Lpb. plantarum* genomes obtained from the NCBI database^2^. Among the publicly available genomes, *Lpb. plantarum* WCFS1 was selected and used as a reference genome (Supplementary Table 1). Comparative genome analysis was performed using bi-directional BLASTp alignments with a cutoff *E*-value of 0.0001, with at least 50% amino acid identity across a minimum of 50% of protein length. The cluster of orthologous genes across strains was defined using the mclblastline algorithm (van Dongen and Abreu-Goodger, 2012).

Pan-genome analysis and visualization were performed using Anvi’o platform v6.1 implemented in Bioconda^3^, using the nucleotide sequence of the 42 *Lpb. plantarum* genomes as input for the microbial pangenomic workflow as described in Anvi’o tutorial^4^. Furthermore, a one-to-one BLASTp (Altschul et al., 1990) comparison of our three novel *Lpb. plantarum* strains against the reference strain WCFS1 was performed to identify the homologous genes and identify potential probiotic features (threshold of 50% of identity across 50% of protein length of significative alignments of *E*-value < 0.0001). Genome synteny was explored within the genomes using nucleotide sequence dotplots generated by Gepard v1.40 (Krumsiek et al., 2007). A graphical representation of the genetic features and the overall genome organization was visualized using Artemis DNAplotter (Carver et al., 2009).

IslandViewer4 (Bertelli et al., 2017) was the platform used to investigate genomic islands (GIs) in the *Lpb. plantarum* strains, with WCFS1 as the reference strain used to perform the alignment. Both methods, IslandPath-DIMOB and SIGI-HMM, were applied. BAGEL4 software was used to predict the bacteriocin clusters and their organization (van Heel et al., 2018).

The Comprehensive Antibiotic Resistance Database^5^ (CARD) and ResFinder-3.2^6^ (Zankari et al., 2012) were used to analyze antimicrobial resistance genes (AMR) and the resistome, respectively.

Prediction of CRISPR (clustered regularly interspaced short palindromic repeats) sequences, and prophages were obtained *via* CRISPRFinder^7^ and PHASTER webserver^8^, respectively (Zhou et al., 2011; Arndt et al., 2016). Prophage annotations were manually refined against the HHpred database (Zimmermann et al., 2018). Moreover, superinfection exclusion (Sie) proteins were predicted manually using the following criteria: a small protein (∼160 amino acids in length) with an N-terminal transmembrane domain detected with TMHMM Server, v. 2.0 (Krogh et al., 2001) and encoded by a gene situated between the integrase- and repressor-encoding gene within the lysogeny module of an identified prophage sequence. Finally, the platform BlasKOALA and Kyoto Encyclopedia of Genes and Genomes (KEGG) mapper were used to predict the metabolic pathways of the strains evaluated^9^ (Okuda et al., 2008; Kanehisa et al., 2019). Approximately half of the genes present on a given genome were assigned to the KEGG families in the three cases (54.7% LT52, 52.1% 513, and 52.4% C9O4).

## Results

### Overview and General Features of the *Lactiplantibacillus plantarum* Strains

**FIGURE 1:**
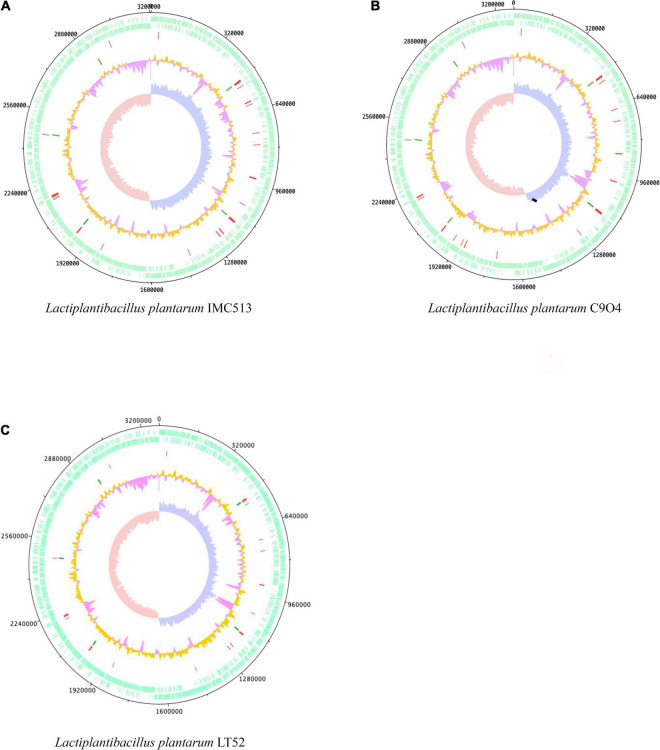
Genome atlases of the three *Lactiplantibacillus (Lpb.) plantarum* strains. **(A)** *Lpb. plantarum* IMC513, **(B)** *Lpb. plantarum* C9O4, **(C)** *Lpb. plantarum* LT52. The three figures are organized from outer to inner circles, circles indicate (i) CDS (+, forward strand), (ii) CDS (–, backward strand), (iii) tRNA (red), (iv) rRNA (green), (v) GC content (orange and pink), and (vi) GC skew (leading strand blue and lagging strand red).

The chromosomal properties of the three *Lpb. plantarum* strains (IMC513, C9O4, and LT52; Figure 1) sequenced in this study are summarized in Table 2. The raw reads assembly resulted in the generation of bacterial chromosomes each with a size similar to that previously reported for sequenced *Lpb. plantarum* isolates (range of 2.8–3.3 Mbp)^2^. The three strains evaluated here possess an average length of 3.251 Mbp, 3,043 coding sequence (CDS), and a GC content of approximately 45%. However, the number of tRNA and rRNA genes vary slightly among the strains. The majority of the genes that appear to be unique for each strain corresponded to hypothetical proteins.

**TABLE 2:** Comparison of chromosomal properties of *Lactiplantibacillus(Lpb.) plantarum* strains.

Strains	IMC513	C9O4	LT52
Genome size (bp)	3,206,211	3,275,253	3,272,989
GC content (%)	45.72	45.57	44.6
Coding sequence (CDS)	3,022	3,069	3,039
tRNA	71	65	66
rRNA	11	11	16

To investigate the chromosomal synteny of the three obtained *Lactiplantibacillus* genomes, whole-genome nucleotide alignments were performed and represented as a dotplot matrix (Supplementary Figure 1), using *Lpb. plantarum* WCFS1 as a reference. The alignment was performed using the *dnaA* chromosomal replication initiation gene as the starting point on the forward strand for each genome. Genome synteny was well conserved in each of the *Lactiplantibacillus* strains providing confidence that the draft genomes were correctly assembled and orientated (Supplementary Figure 1).

### Comparative Genomic Analysis

**FIGURE 2:**
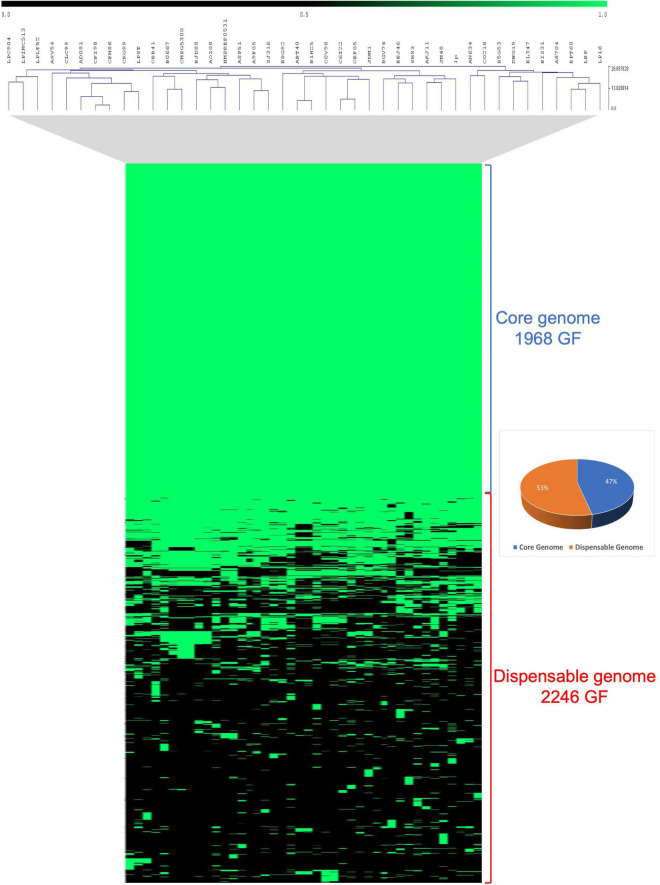
Comparative analysis of 42 *Lpb. plantarum* strains. Comparative analysis realized in Tmev viewer and representing the presence/absence of gene families (GF) from the core and dispensable genomes of 42 *Lpb. plantarum* strains. Presence is indicated in green while absence is indicated in black.

The genomic diversity of three novel *Lpb. plantarum* strains together with 39 previously published *Lpb. plantarum* isolates from a large spectrum of niches was investigated^2^ (Supplementary Table 1). Comparative analysis of these 42 *Lpb. plantarum* genomes was performed using an all-against-all reciprocal BLASTP analysis followed by mclbastline clustering using a previously described method (van Dongen and Abreu-Goodger, 2012; De Angelis et al., 2014). The comparative analysis returned a total of 4,214 orthologous gene families (GF) present across 42 strains, of which 1,968 (47%) represent the core genome and are present in at least one copy across all assessed strains, while 2,246 (53%) constitute families containing accessory gene functions only present in some strains (Figure 2).

**FIGURE 3:**
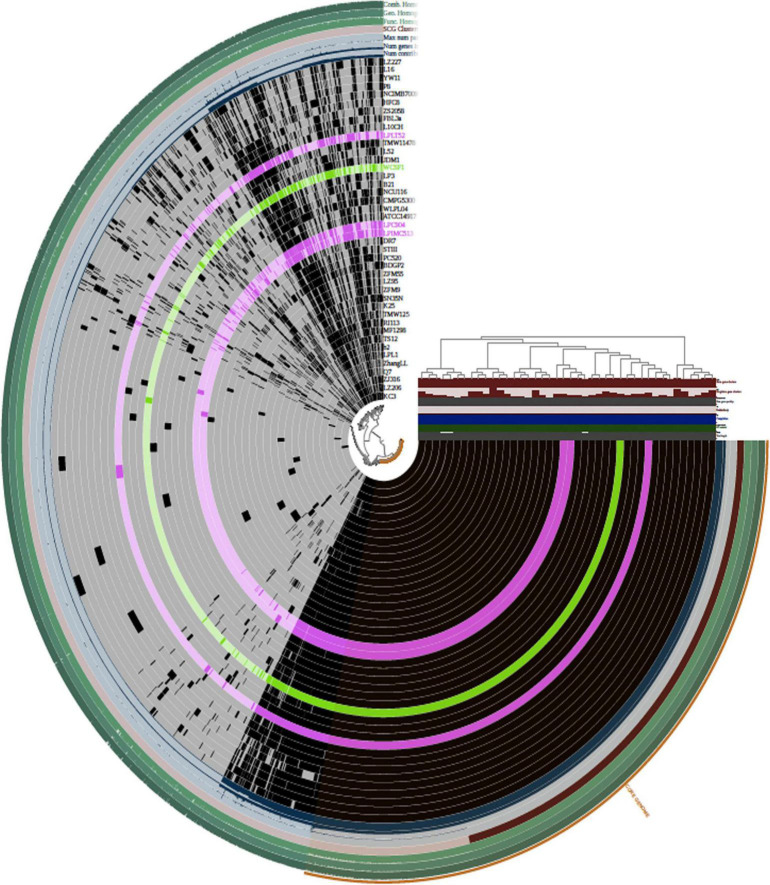
Hierarchical clustering (HCL) of OGs based on their presence/absence across the strains as computed using Anvi’o package (Bioconda). Each ring represents an *Lpb. plantarum* strain. *Lpb. plantarum* WCFS1 is represented in green and the three novel *Lpb. plantarum* strains are represented in pink. The top right panel reports additional information about the dataset (number of gene clusters, singleton gene clusters, number of genes per kbp, redundancy, degree of completeness, GC content, and total length of the genome). The strain clustering tree refers to strain grouping based on the distribution of OGs.

Pan-genome analysis and visualization were performed using the Anvi’o platform on the total coding genetic content predicted for the 42 *Lpb. plantarum* genomes (Figure 3). Hierarchical clustering (HCL) dendrogram obtained using Anvi’o platform and based on the presence/absence of GF and Euclidean distance divided the 42 *Lpb. plantarum* strains into six main clades (identified as Clades 1–6), with the three strains sequenced in this study being members of either clade 4 (in the case of strains C9O4 and IMC513) or Clade 5 (strain LT52) (Figure 3). As a result of the analysis, two major groups were identified. The first one contained only six strains of which four were dairy isolated. The other main group contained the remainder of the strains. The three strains sequenced in the article belonged to the second group, which can be divided into five subgroups where no strain groupings could be correlated with the origin of isolation.

### Genomic Islands

Genomic islands in bacterial genomes are known to carry genes that offer selective advantages to the host. Their prevalence is often the result of external environmental pressures, and they help in aiding our understanding of the relationship between genetic factors and host phenotype. The predicted GIs are displayed in genome plots (Supplementary Figure 2). The analysis revealed the presence of 25 putative GIs for IMC513 raging in size from 4 to 55 kbp. A number of these regions contained no predicted functions which could be used to characterize their origin. Two regions were found to be integrated prophage, one region was predicted to be an integrated plasmid based on the presence of a plasmid mobilization protein (MobA) and a predicted toxin/antitoxin system. Interestingly, this region also contained a predicted heavy metal resistance operon. A 25 kbp region was also identified containing a putative oligopeptide uptake system, *Opp* operon with similarity to that of *Lactococcus lactis* which may indicate it was horizontally acquired. *Opp* is a transporter operon belonging to the ABC transporter superfamily, which mainly transports di-, tri-, and oligopeptides (Charbonnel et al., 2003). The predicted operon was composed of five genes which encode: a dipeptide binding protein (OppE), two integral hydrophobic membrane proteins (OppB and OppC), and two nucleotide-binding proteins (OppD and OppF). This operon was also identified in strains C9O4 and LT52.

A total of sixteen putative GIs were identified in the C9O4 genome, ranging in size from 4 to 55 kbp. Two regions were found to be integrated prophage and one region was predicted to be an integrated plasmid based on the presence of *rep*B (plasmid replication initiation), *mob*A, and a plasmid conjugation operon. Moreover, two predicted integration regions contained a large number of genes related to exopolysaccharide (EPS) production.

Finally, 16 putative GIs were identified in LT52 ranging from 5 to 57 kbp in size with similar predicted functions to the two previous strains. In this instance, however, an incomplete operon involved in the metabolism of myo-inositol was detected. The structural organization of the *Lpb. plantarum* LT52 operon follows the same pattern as in the previously described clusters (Yebra et al., 2007). The cluster harbors four inositol 2-dehydrogenase (*iolG*) genes, one inosose dehydratase (*iolE*), and one sugar transporter gene. Moreover, as for the other two strains sequenced in this work, a plantaricin operon was also predicted in the genome of the *Lpb. plantarum* LT52 (Supplementary Figure 2), which may be taken up as a defense mechanism. A full genomic dissection of this operon is performed in section “Bacteriocin Production”.

### Mobilome and Resistome

**FIGURE 4:**
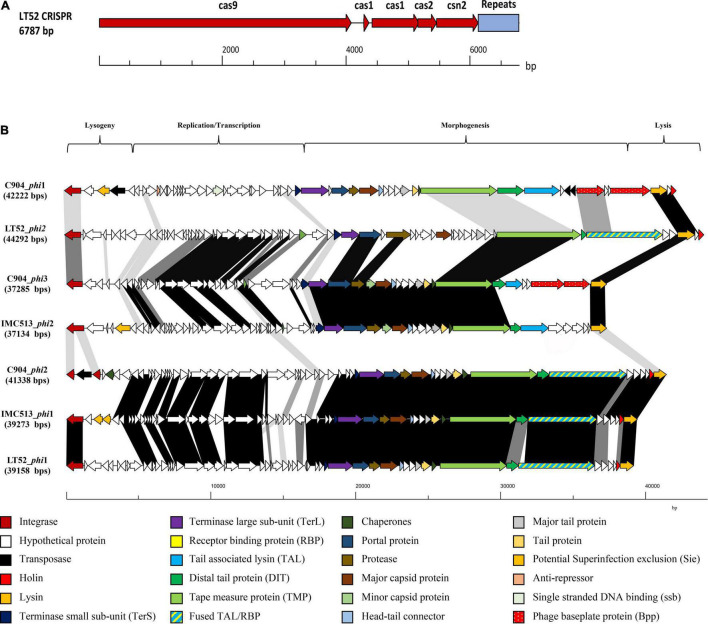
**(A)** Gene map of the genetic organization of the CRISPR 2 loci in *Lpb. plantarum* LT52. **(B)** Comparative analysis of prophage in *Lpb. plantarum* IMC513, C9O4, and LT52 strains. Gene maps show complete prophage regions from lysogeny to lysis regions, arrows representing ORF colored according to predicted function. Shaded boxes correspond to percentage amino acid identity between ORFs.

It has been previously demonstrated that CRISPR and their associated *cas* genes provide resistance against invasive mobile genetic elements (Barrangou et al., 2007; Crawley et al., 2018). Structurally, these systems are identified by a genetic locus encoding a CRISPR repeat-spacer array and *cas* genes. Although the incidence of CRISPR-cas9 is relatively common in bacteria, only half of all sequenced bacterial genomes harbor them. Previous studies showed that, among the 165 strains of *Lpb. plantarum* evaluated, only 26 had CRISPR systems, and of those, 12 contained a type II system (Crawley et al., 2018). In this study, it was found that only one of the three evaluated strains, *Lpb. plantarum* LT52, contains a predicted type II CRISPR-Cas system, with four *cas* genes (*cas*9, *cas*1, *cas*2, and *cns*2). The general features of the CRISPR system are summarized in Figure 4A and Table 3.

**TABLE 3:** Genomic features of CRISPR-Cas system in *Lpb. plantarum* LT52.

Gene	Genome position start	End	Orientation
Csn2_0_IIA	108,839	112,915	+
Cas2_0_I-II-III	113,250	114,014	+
Cas1_0_II	113,992	114.297	+
Cas9_0_II	114,294	114,971	+

Clustered regularly interspaced short palindromic repeats systems target protospacer sequences stored in the CRISPR array to provide immunity against previously encountered phages and plasmids (Table 4). BLAST-based analysis of the CRISPR spacer sequences in LT52 revealed three different spacers targeting *Lactobacillus dionysus* phage. In addition, another four spacers were found against putative prophage or mobile elements that have not yet been characterized (Supplementary Table 2).

**TABLE 4:** Analysis of CRISPR II system direct repeats.

Start	114,996
End	115,559
DR consensus	GTCTTGAATAGTAGTCATAT CAAACAGGTTTAGAAC
DR length	36
Spacers count	8
Direction	+

The PHASTER server was employed to predict the presence of prophage sequences in each of the three evaluated strains (Table 5). BLASTp and HHpred (Zimmermann et al., 2018) were used to further evaluate the homology of these prophages (Figure 4B). The BLASTp analysis revealed three distinct genetic lineages within the prophage sequences based on the prophage structure encoded by the morphogenesis region. Remarkably, while gene synteny and function were highly conserved, amino acid identity was not conserved between the groups. The first lineage represented by strain C904 prophage 1 encodes an apparent complex baseplate structure specified by two large *bppU* homologs (Legrand et al., 2016). These were also predicted in the C904 prophage 3 but with low amino acid identity to those present in C904 prophage 1. C904 prophage 3 shared the highest similarity with IMC513 phage 2. The final lineage contained three closely related prophages defined by a fused TAL/RBP structure. Interestingly, each of the three *Lpb. plantarum* strains in this study are represented in this group, especially considering the diverse ecological niches from which they were isolated. This suggests a common *Lpb. plantarum* ancestor from which the prophage sequence was passed on, or a prolific prophage family infecting *Lpb. plantarum*. Considering that phages are ubiquitous within the environment and guided by host specificity, it is probable that they will target multiple environmental niches.

**TABLE 5:** PHASTER analysis of the three *Lpb. plantarum* strains.

*Lpb. plantarum* strain	Predicted prophages	Length bps	Predicted complete
IMC513	Phage 1	39,273	YES
	Phage 2	37,134	YES
	Phage 3	44,332	NO
	Phage 4	18,915	NO
C9O4	Phage 1	42,222	YES
	Phage 2	41,338	YES
	Phage 3	37,285	YES
LT52	Phage 1	39,158	YES
	Phage 2	44,292	YES

Superinfection exclusion proteins represent a phage resistance system since they prevent DNA injection by certain phages (Mahony et al., 2008). Interestingly, one Sie (IMC513_phage2), and a further three potentially novel Sies’ were identified in *Lpb. plantarum* IMC513 and C9O4, according to the criteria of a small protein (<25 kDa) encoded by a gene situated between the integrase and repressor genes and possessing one or more N-terminal transmembrane domain(s).

### Antibiotic Resistance Genes

The over-prescription and excessive use of antibiotics in both human health and animal agriculture have pressured microbes to acquire resistance genes that allow them to survive in an antibiotic-environment. As these genes may be horizontally transferred, which may have a significant impact on the treatment of bacterial infections, it is of paramount importance to not introduce them to the food chain. Consequently, probiotic bacteria which are often taken to replenish the microbiome after antibiotic treatment should be free from loci encoding potential for antibiotic resistance genes from a clinical safety perspective (Imperial and Ibana, 2016).

As reported in the literature, *Lpb. plantarum* strains generally do not harbor AMR genes, however, partial hits against putative genes annotated as antibiotic resistance genes are sometimes encountered (Evanovich et al., 2019). The analysis of AMRs using two different databases, CARD and ResFinder-3.2, did not indicate potential antibiotic resistance for any of the three strains evaluated. Although those data should be followed by phenotypic testing in terms of clinical decision-making, WGS-deduced AMR profiles can serve as a first screening given the strong correlation between the phenotypic and genotypic AMR profiles as previously shown (Do Nascimento et al., 2017).

### Probiotic Properties

Comparative analysis showed that around 47% of the *Lpb. plantarum* WCFS1 genes are shared by the three *Lpb. plantarum* strains investigated. Among these genes, this study focused our research on those conferring putative probiotic properties. A probiotic bacterium should have the ability to survive and transiently persist in the gastrointestinal tract, where it has to be able to exert a beneficial effect.

### Bacteriocin Production

Bacteriocins are antimicrobial peptides commonly produced by bacteria that possess antimicrobial properties inhibiting the growth of different microorganisms. Due to their antimicrobial activity, they have attracted interest as natural preservatives in the food industry.

**FIGURE 5:**
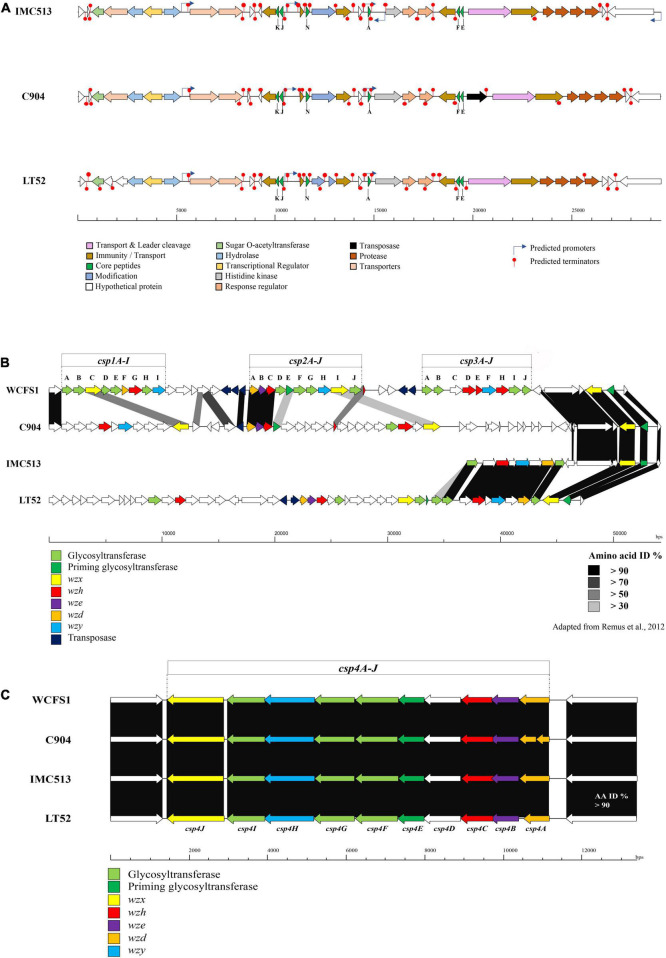
**(A)** Overview of plantaricin operons in the three *Lpb. plantarum* evaluated. Gene maps show complete plantaricin regions with arrows representing ORF colored according to the predicted function. Bagel3 predicted promotors and terminators are also indicated. **(B)** Genetic organization of the putative polysaccharide biosynthesis gene clusters (*cps*) of the three *Lpb. plantarum* strains evaluated. **(C)** Map of the *Lpb. plantarum* WCFS1 cps gene clusters 1, 2, and 3 and comparison with the *Lpb. plantarum* strains C9O4, IMC513, and LT52.

The screening of the entire genome sequences of three *Lpb. plantarum* isolates revealed that a bacteriocin encoding locus (*pln* locus) was located in a 29 kbp long region organized in an operon-like structure conserved in all three strains. The three strains evaluated encode genes for two peptides, plantaricin *plnJ*K (class IIb) *pln*EF (class I), previously described for other *Lpb. plantarum* strains (Goel et al., 2020). Moreover, the *pln* locus was also found to contain *pln*A described in other *Lpb. plantarum* strains such as C11, WCFS1, and V90, and another bacteriocin-like gene *pln*N (Figure 5A). The locus also contains the genes *pln*H and *pln*G, which encode an ABC transport system also confirmed in other plantarum strains (Rizzello et al., 2014; Goel et al., 2020). All identified plantaricin genes encode a primary amino acid sequence, which includes an N-terminal amino acid leader peptide followed by an amino acid core peptide. The leader peptide was shown to end in the double glycine (GG) motif reported for many bacteriocins produced by Gram-positive bacteria (Dirix et al., 2004; Flórez and Mayo, 2018). All bacteriocins presented in the three strains showed 100% homology to each other (Table 6).

**TABLE 6:** Amino acid sequence of the plantaricin encoding genes belonging to the *pln* locus.

*pln* genes	Function	Amino acid sequence
*pln*JK	Prebacteriocin with GG leader	–
*pln*K	Bacteriocin	MKIKLTVLNEFEELTADAEKNISGGRRSRKNGIGYAIGYAFGAVERAVLGGSRDYNK
*pln*J	Bacteriocin	MTVNKMIKDLDVVDAFAPISNNKLNGVVGGGAWKNFWSSLRKGFYDGEAGRAIRR
*pln*N	Bacteriocin	MKSLDKIAGLGIEMAEKDLTTVEGGKNYSKTWWYKSLTLLGKVAEGTSSAWHGLG
*pln*A	Induction pheromone	VIIMKIQIKGMKQLSNKEMQKIVGGKSSAYSLQMGATAIKQVKKLFKKWGW
*pln*EF	Prebacteriocin with GG leader	–
*pln*F	Bacteriocin	MKKFLVLRDRELNAISGGVFHAYSARGVRNNYKSAVGPADWVISAVRGFIHG
*pln*E	Bacteriocin	MLQFEKLQYSRLPQKKLAKISGGFNRGGYNFGKSVRHVVDAIGSVAGIRGILKSIR
*pln*G	ABC transporter	–
*pln*H	Immunity protein	–

### Exopolysaccharide Production

The production of EPSs by LAB has implications from the perspective of both technological and health aspects. Technologically, previous studies have correlated the production of EPSs with improving the rheological and texture properties of fermented products, especially in dairy products. Moreover, EPS is involved in phage absorption, adhesion to human cells (Lee et al., 2016), and exhibiting antitumor properties (Wang et al., 2008). There is a high diversity among EPS clusters identified in *Lactobacillus* strains (Deo et al., 2019), especially within the *Lpb. plantarum* species. Up to five different EPS clusters have been described for *Lpb. plantarum* strains. This is consistent with our results, as none of the three strains evaluated share the same EPS cluster (Figures 5B,C). According to a study published by Remus et al. (2012), four gene clusters associated with EPS production have been described in *Lpb. plantarum* WCFS1. These four gene clusters could be divided into two main groups. The first group encodes the majority of functions required for capsular polysaccharide formation (2A-J and *cps*4A-J), while the other two (*csp*1A-I and *cps*3A-J) were predicted to lack genes encoding chain-length control functions and priming glycosyl-transferases (Remus et al., 2012). However, not all *Lpb. plantarum* strains evaluated possessed the four clusters of EPS genes. *Lpb. plantarum* strains ST-III and the type strain ATCC14197 only possess the csp3 and csp4 clusters, while JMD1 only carries the cps4 cluster. This variability between the strains is reflected also in the location of the clusters, encoded within genomic recombination hotspots and prone to horizontal gene transfer. In this study, the genome of *Lpb. plantarum* WCFS1 was used as a model to predict the EPS clusters in the three evaluated *Lpb. plantarum* strains as it contains the four typical *Lpb. plantarum* EPS clusters and is well characterized (Remus et al., 2012). In WCFS1, a region of approximately 49 kbp contains three of the four gene clusters (*cps*1A-I to *cps*3-J), all of them separated by transposon genes. The fourth region (approximately 14 kb), encodes the *cps*4 gene cluster. It was previously reported that this cluster is the most conserved in *Lpb. plantarum* strains and this is also the case among the strains in this study (Figure 5C). The amino acid identity between each of the four strains evaluated was higher than 90% across the entirety of cluster 4 with each of the strains encoding the complete operon (*cps*4A-J). The first three genes (*cps*4ABC) encode a tyrosine kinase phosphor-regulatory system (also named wzd, wze, and wzh). The gene *cps*4D is predicted to encode a UDP-N- acetylglucosamine 4-epimerase. The fifth gene *cps*4E encodes the priming glycosyltransferase, *cps*4J a flippase (wzx), *cps*4H a polymerase (also named wzy), and the last three (*cps*4FGI) present high similarity to glycosyltransferase genes.

The examination of the region which encodes clusters 1–3 showed a significant level of variation between the strains (Figure 5B). *Lpb. plantarum* IMC513 encodes only cluster *csp*3, which appears to be highly conserved with the *csp*3 in LT52, although the glycosyltransferases encoded by these strains are divergent to those of WCFS1 and may indicate a different sugar coating on the cell wall. Cluster 3 (*cps*3A-J) in *Lpb. plantarum* WCFS1 was predicted to be involved in the synthesis of a polysaccharide made up of acetylated quatro-saccharide repeating units (Jiang and Yang, 2018). *Lpb. plantarum* LT52 appears to contain both *csp*3 and *csp*2, however, the glycosyltransferases of *csp*2 do not share any similarity to those of *Lpb. plantarum* WCFS1. Finally, *Lpb. plantarum* C9O4 contains *cps*2 and an apparently unique cluster in place of *csp*1. None of the strains appear to encode cluster *cps*1 from WCFS1.

### Host Adhesion Proteins

The analysis of the three genomes allowed for the identification of several genes involved in host adherence. Based on the previous investigation in *Lpb. plantarum* WCFS1, adhesion factors were identified in the *Lpb. plantarum* strains evaluated, containing domains to adhere to collagen, chitin, fibronectin, and mucus (van den Nieuwboer et al., 2016; Table 7). In particular, mucus-binding proteins (lp_3114, lp_3059 and lp_1643) were found in the three strains. Interestingly, lp_1229, which encodes a Msa (mannose-specific adhesion) protein, and for which deletion has been shown to result in a loss of the ability to agglutinate with yeast, is not present in LT52.

**TABLE 7:** Prediction of genes related to host–microbe interactions.

Predicted function gene	Locus tag C9O4	Locus tag IMC513	Locus tag LT52	Locus tag WCFS1
Fibronectin/fibrinogen binding protein	lpC9O4_1481	lpIMC513_1565	lpLT52_1565	lp_1793
LPxTG cell wall anchor	lpC9O4_2172	lpIMC513_2180	lpLT52_2166	lp_2486
Mannose specific adhesion	lpC9O4_1051	lpIMC513_1079	–	lp_1229
Mucus-binding protein	lpC9O4_2681	lpIMC513_2684	lpLT52_2666	lp_3114
	lpC9O4_2634	lpIMC513_2637	lpLT52_2619	lp_3059
	lpC9O4_1615	lpIMC513_1429; lpIMC513_1430	lpLT52_1437	lp_1643
chitin-binding protein	lpC9O4_1564	lpIMC513_1481	lpLT52_1480	lp_1697

Moreover, the three stains also encode proteins with an LPxTG motif, a cell wall anchor domain that can be covalently anchored to peptidoglycan *via* sortase activity and is involved, among others, in eukaryotic host cell adhesion (Desvaux et al., 2006). LysM motifs domains and peptidoglycan binding motifs were also found in the *Lpb. plantarum* strains. They are known to promote cell wall association and allow cell immobilization (Visweswaran et al., 2014). A chitin-binding gene (lp_1697) and fibronectin/fibrinogen-binding genes were also identified.

### Sugar Import and Central Carbon Metabolism

The analysis of the metabolic capabilities of each of the three strains in this study was carried out against the KEGG database. Sugar transport and metabolism genes were annotated manually. On average, approximately 11% of the identified genes in each of the three *Lpb. plantarum* genomes were found to be involved in carbohydrate metabolism, which is similar to the most studied *Lpb. plantarum* strain WCFS1. The higher number of genes encoding putative sugar transports is the main reason for its versatility and ability to grow on a wide variety of sugar sources. The majority of these transporters are predicted PEP-dependent sugar PTS, as has been described for other *Lpb. plantarum* strains (Kleerebezem et al., 2003; Zhang et al., 2018). The three analyzed *Lpb. plantarum* strains harbor around 300 genes related to transport. Among them, the majority are PTS and ABC. In total, the three *Lpb. plantarum* possess 16 complete PTS enzyme II complexes and several incomplete complexes.

In addition to PTS, the genome of the three *Lpb. plantarum* was shown to encode other transporter systems that were predicted to be involved in the transport of carbon sources, the ABC-system. Around a 100 genes are predicted to be involved in the ABC transport systems. These ABC sugar systems can import more than one substrate and expand the carbon transport capacity of *Lpb. plantarum* (Zhang et al., 2018). It has previously been shown that WCFS1 (among other *Lpb. plantarum* strains) encodes a large variety of proteins involved in sugar uptake and utilization which may help niche colonization and adaptation (Siezen et al., 2012).

The analysis by BlastKOALA indicated differences in carbohydrate utilization between the three strains evaluated. C9O4 and IMC513 possess genes for ten carbohydrate pathways, including the Embden–Meyerhof pathway (EMP), pentose phosphate pathway (PP) (both oxidative and non-oxidative), galactose degradation pathway (Leroir pathway), while LT52 only possesses the incomplete oxidative phase of the PP and the EMPs. Moreover, the pathways related to gluconeogenesis, glycogen degradation, and Enter–Doudoroff pathways also appear to be incomplete according to the analysis.

### Vitamins and Cofactors Biosynthesis

Riboflavin, also known as vitamin B2, is a water-soluble vitamin considered a central component of cellular metabolism since it is the precursor of other coenzymes and thus essential for the energy metabolism of the cell (Mohedano et al., 2019). The genomes of the three *Lpb. plantarum* strains evaluated revealed a potential operon containing some of the genes involved in the synthesis of riboflavin. The operon contains GTP cyclohydrolase II (ribA), the riboflavin synthase alpha chain (ribB), 5-amino-6-(5-phosphoribosylamino) uracil reductase (ribD), riboflavin synthase (ribE), riboflavin kinase (ribF), 6,7-dimethyl-8-ribityllumazine synthase (ribH), and other related enzymes. Moreover, all enzymes required for the biosynthesis of Coenzyme A from (R)-pantothenate are also present in the three strains. However, the enzymes involved in the biosynthesis of the molybdenum cofactor are only presented in LT52. Finally, one block is missing in the tetrahydrofolate, L-threo-Tetrahydrobiopterin, and tetrahydrobiopterin biosynthesis in all the strains evaluated.

### Stress-Related Genes and Bile Acid Metabolism

According to the definition of probiotics, a probiotic strain when consumed has to overcome different stresses to survive in the gastrointestinal tract where it then exerts beneficial properties. The ability to resist host-imposed stressful conditions or to express bile salt hydrolase genes are key factors to consider when searching for potential candidate probiotic bacteria. *In silico* analysis of the three strains demonstrates that *Lpb. plantarum* encodes genes for several stress-related proteins. In particular, stress related with temperature, acid tolerance, and osmotic pressure were evaluated.

As LAB, *Lpb. plantarum* possess the ability to survive acidification of their local environment, which serves also as an advantage in overcoming the pH of the stomach to which they are exposed when consumed. The F0F1-ATPases (subunits A, C, delta, alpha, gamma), present in all strains, have been identified as major regulators of intracellular pH. Moreover, sodium-proton antiporters (9 for LT52 and 11 for C9O4 and IMC513) and alkaline shock proteins are identified in the three strains and have been proven to be involved in the response to acid stress.

The three *Lpb. plantarum* isolates encode the GroES-GroEL chaperonin and the HrcA-DnaK-DnaJ-GrpE operon, which encode heat shock proteins. Moreover, the strains also encode small heat shock proteins of the HSP20 family, which have been shown to affect membrane fluidity (Capozzi et al., 2011). Finally, three highly homologous cold-shock proteins (Csp) were identified.

The osmoregulatory system *opu* glycine betaine/carnitine/choline ABC transporter, that consists of the genes (*opu*ABCD), was also identified in each of the *Lpb. plantarum* strains evaluated.

To overcome oxidative stress, all evaluated strains possess genes related to glutathione peroxidase, four NADH oxidases, and two NADH peroxidases (in the case of LT52 only one NADH peroxidase). Thiol peroxidase is present in IMC513 and C9O4 but not in LT52. Moreover, thiol exporters (CydC and CydD), manganese transporters, and metal transporters were also identified in the three strains. Finally, the energy-dependent proteases, lon, ClpP, and HslV, closely involved in stress response, were also identified (Kleerebezem et al., 2003).

The analysis of the genomes revealed that all the *Lpb. plantarum* strains evaluated possessed four genes related to bile salt hydrolase activity, namely *bsh*1, *bsh*2, *bsh*3, and *bsh*4, that were previously identified for *Lpb. plantarum* WCFS1 (Lambert et al., 2008). The nucleotide identity between each of the four strains evaluated was higher than 95% across the four *bsh* genes when compared with the ones described for *Lpb. plantarum* WFS1. Functional bile salt metabolism by all evaluated strains was previously performed by Prete et al. (2020). The deconjugation strength of the isolates to generate free bile acids was higher for LT52, followed by C9O4 and IMC513.

## Discussion

This study reports the draft genome sequence of three *Lpb. plantarum* strains, IMC513, C9O4, and LT52, isolated from the gastrointestinal tract, table olives, and raw-milk cheese, respectively, with insights into the potential probiotic properties of these strains based on the presence/absence of putative beneficial genes. Importantly, the food-dwelling *Lpb. plantarum* strains analyzed are representative of isolates that are naturally consumed at very high levels (∼10^8^ per gram) in table olives or (∼10^7^ per gram) cheese (Perpetuini et al., 2020), and it is, therefore, important to understand the genetic makeup of these strains and their potential impact on the host. Through this genomic analysis, we aimed to obtain insights into the key genes and predict the functionality of these strains to underpin future phenotypic and technological studies (Garcia-Gonzalez et al., 2018, 2021).

The genetic relatedness between the strains and 39 complete sequences of *Lpb. plantarum* was evaluated by phylogenetic analysis. First, the link between strain origin and gene content was evaluated by analyzing the orthologous GF. The result of this analysis highlighted two main findings: first, there is significant diversity among the *Lpb. plantarum* strains, since 53% of the GF evaluated corresponded with the variable genome, and second, no origin of isolation-dependent grouping was found among the strains. In particular, the three strains evaluated were placed in the same clade, even though they were isolated from three different sources. These findings are in line with those described by Martino et al. (2016), who evaluated 54 strains isolated from different habitats to explore the link between intra-species genetic variability and their environmental origin. Using a different approach (HCL dendrogram based on presence/absence of GF and Euclidian distance), they divided the *Lpb. plantarum* strains into two major clades, with six different sub-clades. Strains IMC513 and C9O4 both appeared inside the same clade (4) together with ATCC 14917, DR7, and WLPL04, isolated from pickled cabbage, dairy products, and human sources, respectively. LT52 belonged to a larger clade (5), which also includes strains isolated from different sources, including human isolates, vegetables, and dairy products. The closest strains related to LT52 are L10CH and TMW11478, isolated from dairy products and honey, respectively. The fact that the *Lpb. plantarum* strains are not highly specialized for particular niches, potentially *via* the presence of adaptation islands, indicates that *Lpb. plantarum* is an extremely versatile species.

The detection of multiple GIs within the *Lpb. plantarum* strains analyzed is evidence of horizontal gene transfer. The acquisition of such islands increases the adaptability of these strains by allowing them to adapt to various niches and stresses. This is evidenced by the presence of the *Opp* operon, involved in oligopeptide uptake for lactose utilization found in the three strains, and with similarities to that of *Lactococcus lactis*. This, combined with the low number of shared orthologous groups (OGs) (<50%) within the isolates screened, would seem to demonstrate a high level of genome plasticity and flexibility within the plantarum species. Moreover, the *Lpb. plantarum* genomes evaluated display a large number of genes related to carbohydrate metabolism, which allow *Lpb. plantarum* to grow on a large variety of carbohydrate sources and inhabit a wide variety of niches. As it has been reported, the high variability of sugar intake related to *Lpb. plantarum* is an advantage that is not present in other *Lactobacillus* species sequenced. As LAB, *Lpb. plantarum* possess the mechanisms to survive in low pH environments, mainly attributed to the presence of F0F1-ATPases, which are important as mechanisms of survival but also for their applicability as starter cultures in the food industry or as probiotics (Siezen et al., 2012).

While the two food strains tested are consumed in high quantities in certain foods, they may have the potential for development as potential food additives (probiotics). The status of these strains as potential probiotic bacteria is supported by their safety status and the procession of a set of genes which can confer potential probiotic traits. According to the definition of a probiotic, to consider a strain a potential probiotic it must survive the gastrointestinal tract environment, transiently persist, and exert beneficial properties on the host (Hill et al., 2014). Adhesion to host cells has been reported as being one of the key criteria for probiotic strains to exert beneficial effects on the host (Hill et al., 2014). The presence of mucus-binding proteins, other surface anchor proteins, and the production of EPSs allow these *Lpb. plantarum* strains to adhere to other bacteria, extracellular matrix, or host cells. This confirms the previously carried out results, in which the three *Lpb. plantarum* strains evaluated showed a marked adhesion efficiency to both the mucus and intestinal cells (Garcia-Gonzalez et al., 2018). We suggest that the survival of the strains in the gastrointestinal tract appears to be likely due to the presence of the genes related to stress tolerance that are common to both human- and environmental-adapted strains (Lebeer et al., 2008). Such inferences have been made based on previous analyses showing the adaptation of the strain WCFS1 to stressful environments (Siezen et al., 2012). In particular, the three strains evaluated are likely to survive in a high-osmolarity environment, thanks to the presence of the osmoregulatory system *opu* (Kleerebezem et al., 2003). The presence of other beneficial traits such as antioxidant-related genes and bacteriocin production, along with the similarity to human gut strains, reinforces the likelihood that these strains may survive in the GI tract. Furthermore, many of these properties (including EPS production and BSH activity) have been associated with microbe–host signaling, and therefore, may have the potential to directly impact the host physiology (Joyce et al., 2014; Ryan et al., 2019). This will be evaluated through further studies by our group.

The presence of natural mechanisms of phage resistance is also of interest if these strains are to be developed for industrial fermentations. Among the natural resistance mechanisms found in the *Lpb. plantarum* strains evaluated, it is notable to highlight the presence of a complete CRISPR-Cas system and Sie proteins. Interestingly, a complete CRIPSR system was only found in strain LT52. In particular, the *Lpb. plantarum* strain LT52 possesses the CRISPR-Cas system (type II), with four *cas* genes (*cas*9, *cas*1, *cas*2, and *cns*2). Although type II systems are the most widely distributed among lactobacilli, only 12 of the 165 *Lpb. plantarum* strains analyzed by Crawley et al. (2018) and colleagues appeared to display the system. Sie’s, produced by the resident prophages, are known to inhibit the injection of viral DNA into the bacterial host. One Sie (IMC513_phage2), and a further 3 potentially novel Sie’s were identified in *Lpb. plantarum* IMC513 and C9O4. Interestingly, each strain evaluated in this study has evolved different mechanisms to avoid phage infection, but none of the strains possess more than one such mechanism.

## Conclusion

This study reported the complete genome sequences of three *Lpb. plantarum* strains isolated from different sources, raw-milk cheese, table olives, and the gastrointestinal tract. The results obtained in this study, in association with the previous *in vitro* works performed, highlight the potential of food-dwelling *Lpb. plantarum* strains as probiotics, which appear at very high levels in table olives and cheese. The genome sequence gives us a basis to further elucidate the functional mechanisms of the potential probiotic properties of these strains. The phylogenetic analysis demonstrated that the strains were similar to those isolated from human, environmental and food sources reflecting a lack of niche adaptation. The genomic analysis of the strains revealed the presence of putative genes that may enhance gut survival and future technological development of these isolates as probiotics, including genes related to environmental adaptation, cell adhesion, and stress tolerance. In particular, the presence of the genes associated with the adaptation to gastrointestinal stress and a potential capacity to use a large variety of carbon sources demonstrates the ability of the three *Lpb. plantarum* strains to survive in a wide variety of niches and their potential to be used in biotechnological and pharmaceutical products or as functional agents in foods. Moreover, none of the strains evaluated proved to have antibiotic resistance genes or virulence factors, which suggests their potential safety for such applications.

## Data Availability Statement

The datasets presented in this study can be found in online repositories. The names of the repository/repositories and accession number(s) can be found below: https://www.ncbi.nlm.nih.gov/, PRJNA795971; https://www.ncbi.nlm.nih.gov/, PRJNA795980; https://www.ncbi.nlm.nih.gov/, PRJNA795972.

## Author Contributions

CG and AC developed the study design, coordinated, and supervised the overall study. DS substantially contributed to the conception of the work. NG-G and FB performed bioinformatic analysis. All authors supported the writing of the manuscript, read and approved the final manuscript.

## Conflict of Interest

NG-G undertook an internship at Synbiotec S.r.l. The remaining authors declare that the research was conducted in the absence of any commercial or financial relationships that could be construed as a potential conflict of interest.

## Publisher’s Note

All claims expressed in this article are solely those of the authors and do not necessarily represent those of their affiliated organizations, or those of the publisher, the editors and the reviewers. Any product that may be evaluated in this article, or claim that may be made by its manufacturer, is not guaranteed or endorsed by the publisher.

## Footnotes

1
http://www.efsa.europa.eu

2
https://www.ncbi.nlm.nih.gov/genome/genomes/1108

3
https://anaconda.org/bioconda/anvio

4
http://merenlab.org/2016/11/08/pangenomics-v2

5
https://card.mcmaster.ca

6
https://cge.cbs.dtu.dk/services/ResFinder/

7
https://crisprcas.i2bc.paris-saclay.fr

8
http://phaster.ca

9
https://www.kegg.jp/blastkoala/
