# Supplementary material for: Comparative Genomics of Lactiplantibacillus plantarum: Insights Into Probiotic Markers in Strains Isolated From the Human Gastrointestinal Tract and Fermented Foods
Source: Front Microbiol. 2022 May 18;13:854266. doi: 10.3389/fmicb.2022.854266 (PMC9159523; doi:10.3389/fmicb.2022.854266)
Supplement: Supplementary file 1 [file Data_Sheet_1.docx]

Supplementary Material

# Supplementary Figures and Tables

## Supplementary Figures

**Supplementary Figure 1. Whole genome nucleotide dotplots.** Whole genome nucleotide alignments of three *Lpb. plantarum* strains sequenced in the context of this study and the reference type strain WCFS1.

**
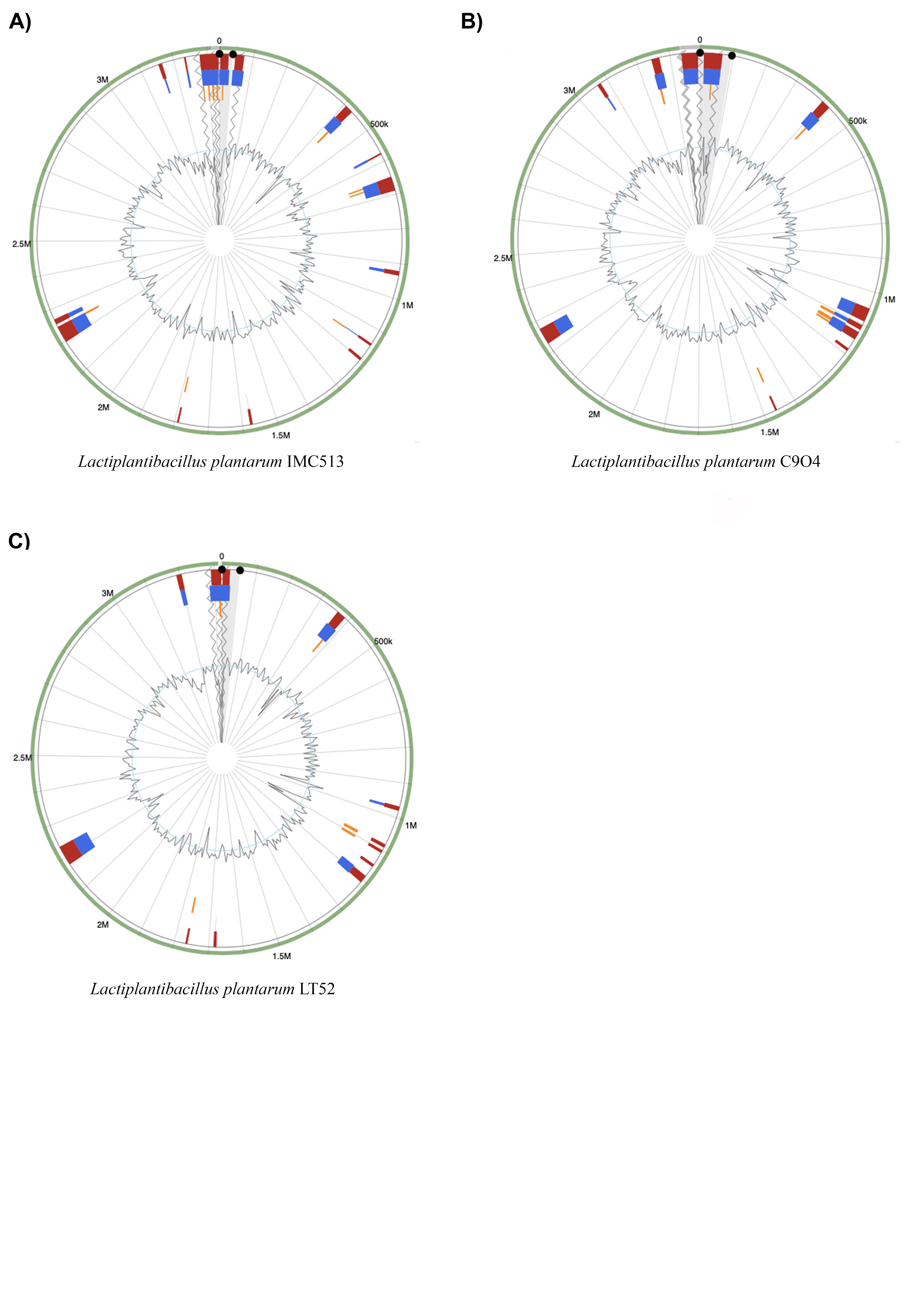
**

**Supplementary Figure 2. Genomic islands predicted in *Lpb. plantarum* IMC513, C9O4 and LT52.** GIs predicted in each of the three genomes are shown in different colors as follows: the entire detected regions in red, IslandPath-DIMOB in blue and SIGI-HMM in yellow. Letters indicate: a) predicted prophages, b) predicted EPS clusters, c) predicted Opp cluster, d) predicted plantaricin cluster, e) predicted myo-inositol.

## Supplementary tables

**Supplementary table 1.** Characteristics of the 42 *Lpb. plantarum* used in this study

| **Strain name** | **Genbank accession** | **Ecological niche** | **Genome Status** | **N. of contigs** | **Citation** |
| --- | --- | --- | --- | --- | --- |
| WCFS1 | AL935263.2 | Human saliva | Complete | 1 | Siezen *et al*., 2012 |
| ZJ316 | CP004082 | Infant faeces | Complete | 1 | Li *et al*., 2013 |
| WLPL04 | LKCO00000000 | Human isolate | Complete | 1 | Tao *et al*., 2015 |
| HFC8 | CP012650 | Human isolate | Complete | 11 | Kumari *et al*., 2015 |
| LZ95 | CP012122 | Infant faeces | Complete | 3 | Li *et al*., 2016 |
| ZFM55 | CP032359 | Infant faeces | Complete | 4 | * |
| ZFM9 | CP032642 | Infant faeces | Complete | 6 | * |
| CMPG5300 | AXZV01000000 | Vaginal isolated | Draft | 48 | Malik *et al*., 2014 |
| IMC513 | n.a | Human isolate | Draft | 25 | ** |
| FBL-3a | CP034694 | Beef cattle faeces | Complete | 1 | * |
| BDGP2 | CP023174.1 | Drosophila melanogaster gut | Complete | 5 | ** |
| P8 | CP005942 | Dairy isolate | Complete | 1 | Tatusova *et al*., 2014 |
| LZ206 | CP015966 | Dairy isolate | Complete | 4 | Li *et al*., 2016 |
| LZ227 | CP015857 | Dairy isolate | Complete | 6 | Li *et al*., 2016 |
| 10CH | CP023728 | Dairy isolate | Complete | 1 | El Halfawy *et al*., 2017 |
| K25 | CP020093 | Dairy isolate | Complete | 7 | Jiang *et al*., 2018 |
| DR7 | CP031318 | Dairy isolate | Complete | 2 | * |
| NCIMB 700965 | CP023490 | Dairy isolate | Complete | 6 | Heeney *et al*., 2019 |
| YW11 | CP035031 | Dairy isolate | Complete | 6 | Wang *et al*., 2015 |
| Q7 | CP019712 | Dairy isolate | Complete | 5 | * |
| LT52 | n.a | Dairy isolate | Draft | 28 | ** |
| RI-113 | CP017406 | Meat Product | Complete | 7 | Inglin *et al*., 2017 |
| TMW 1.25 | CP017354 | Meat Product | Complete | 7 | Kafka *et al*., 2017 |
| MF1298 | CP013149 | Meat product | Complete | 15 | McLeod *et al*., 2019 |
| B21 | CP010528 | Meat product | Complete | 29 | Golneshin *et al.*, 2015 |
| LPL-1 | CP021997 | Fish isolate | Complete | 2 | Wang *et al*., 2018 |
| SN35N | AP018405 | Japanese pear | Complete | 5 | Noda *et al*., 2018 |
| b-2 | CP027349 | Fermented-food pickles | Complete | 1 | * |
| PC520 | CP023772 | Fermented-food pickles | Complete | 3 | * |
| LP3 | CP017066 | Vegetables | Complete | 3 | Jeon *et al*., 2017 |
| C9O4 | n.a | Table olives | Draft | 37 | ** |
| TS12 | CP018324 | Stinky tofu | Complete | 7 | * |
| NCU 116 | CP016071 | Vegetables | Complete | 1 | * |
| ATCC 14917 | NZ_ACGZ00000000.2 | Cabbage pickled | Draft | 39 | Tatusova *et al*., 2014 |
| 5-2 | CP009236 | Fermented soybean | Complete | 1 | Liu *et al*., 2015 |
| ZS2058 | CP012343 | Sauerkraut | Complete | 1 | Yang *et al*., 2015 |
| TMW 1.1478 | CP021932 | Honey | Complete | 2 | Prechtl *et al*., 2018 |
| KC3 | CP025586 | Kimchi | Complete | 4 | * |
| ST-III | CP002222 | Kimchi | Complete | 1 | Wang *et al*., 2011 |
| JDM1 | CP001617 | Grass silage | Complete | 1 | Zhang *et al*., 2009 |
| 16 | NCIMB41875 | Malt production steep water | Complete | 11 | Crowley *et al*., 2013 |
| Zhang-LL | CP011769 | Fermented rice | Complete | 1 | * |

*Unpublished

**Sequenced in the framework of this study

n.a.: not available

**Supplementary table 2.** BLASTn based analysis of CRISPR spacers from *Lb. plantarum* LT52

| **Spacer** | **Sequence** |  | | | | | | |
| --- | --- | --- | --- | --- | --- | --- | --- | --- |
|  |  | **Description** | **Max Score** | **Total Score** | **Query Cover** | **E value** | **Per. Ident** | **Accession** |
| 1 | GACGATGTTCAGAATGATGCTTTAGGGGAA | *Lactobacillus* phage Bromius, Dionysus, lacchus and Semele | 44.1 | 44.1 | 86% | 0.16 | 96.15% | NC_048085.1  MH809530.1  NC_048084.1  NC_047926.1 |
| 2 | TGGTGAACATATCCCGTAAAGTCTGATTGT | *Lactobacillus* phage Bromius, Dionysus, lacchus, Semele, Bacchae and Lpa804 | 48.1 | 48.1 | 93% | 0.010 | 96.43% | NC_048085.1  MH809530.1  NC_048084.1  NC_047926.1  NC_047924.1  NC_048134.1 |
| 3 | CAATTAAGATATATAATTAAGACATTAAAT | *Lactobacillus* phage Dionysus and Lacchus | 60.0 | 130 | 100% | 3e-06 | 100.0% | MH809530.1  NC_048084.1 |
| 4 | AACCATTCGTGGCGTGAGATTGACCGGATC | *Sparus* *aurata* | 40.1 | 72.4 | 66% | 2.4 | 100% | LR537136.1 |
| 5 | TTACTATCTGACTTATTAGAGAATAGATCA | *Ehrlichia muris* AS145 | 40.1 | 40.1 | 66% | 2.4 | 100% | CP006917.1 |
| 6 | AAGGCGACCCCGTTAGCGAACATGGTATTA | ABC transporter-related protein, *Scytonema* sp. HK-05 | 40.1 | 72.4 | 70% | 2.4 | 100.0% | AP018194.1 |
| 7 | TCAATTATTTGATAAAGGACTGTTTATATA | Matrixin, *Acinetobacter sp.* WCHA55 | 42.1 | 42.1 | 70% | 0.62 | 100.0% | CP032286.1 |
| 8 | TATTGATCAGGCCGTGAAGCCTAAGATTTA | *Lactiplantibacillus plantarum* strain Heal19 | 60.0 | 60.0 | 100% | 3e-06 | 100.0% | CP055123.1 |

# References

Crowley, S., Bottacini, F., Mahony, J., and van Sinderen, D. (2013) Complete genome sequence of *Lactobacillus plantarum* strain 16, a broad-spectrum antifungal-producing lactic acid bacterium. *Genome Announc* 1, 4 doi: 10.1128/genomeA.00533-13.

El Halfawy, N. M., El-Naggar, M. Y., and Andrews, S. C. (2017) Complete genome sequence of *Lactobacillus plantarum* 10CH. *Genome Announc* 5, no. 48. doi:10.1128/genomeA.01398-17.

Golneshin, A., Adetutu, E., Ball, A. S., May, B. K., Van, T. T., and Smith, A. T. (2015) Complete Genome Sequence of *Lactobacillus plantarum* strain B21, a bacteriocin-producing strain isolated from Vietnamese fermented sausage Nem Chua.*Genome Announc* 3, 2. doi:10.1128/genomeA.00055-15.

Heeney, D. D., and Marco, M. L. (2019) Complete genome sequence of the plantaricin-sensitive strain *Lactobacillus plantarum* ncimb 700965. *Microbiol Resour Announc* 8, 21. doi:10.1128/MRA.01724-18

Inglin, R. C., Meile, L., Klumpp, J., and Stevens, M. J. A. (2017) Complete and Assembled Genome Sequence of *Lactobacillus plantarum* RI-113. *Genome Announc* 5, 16. doi:10.1128/genomeA.00183-17.

Jeon, S., Jung, J., Kim, K., Yoo, D., Lee, C., Kang, J., Cho, K., *et al*. (2017) Comparative genome analysis of *Lactobacillus plantarum* Gb-Lp3 provides candidates of survival-related genetic Factors. *Infect Genet Evol* 53: 218-26. doi:10.1016/j.meegid.2017.05.015.

Jiang, Y., Zhang, J., Zhao, X., Zhao, W., Yu, Z., Chen, C., and Yang, Z. (2018) Complete genome sequencing of exopolysaccharide-producing *Lactobacillus plantarum* K25 provides genetic evidence for the probiotic functionality and cold endurance capacity of the strain. *Biosci Biotechnol Biochem* 82, 7: 1225-33. doi:10.1080/09168451.2018.1453293.

Kafka, T. A., Geissler, A. J., and Vogel, R. F. (2017) Multiple genome sequences of *Lactobacillus plantarum* TMW 1.25. *Genome Announc* 5, 29. doi:10.1128/genomeA.00654-17.

Kumari, M., Swarnkar, M. K., Kumar, S., Singh, A. K., and Gupta, M. (2015) Complete genome sequence of potential probiotic *Lactobacillus Sp.* Hfc8, isolated from human gut using Pacbio Smrt sequencing. *Genome Announc* 3, 6. doi:10.1128/genomeA.01337-15.

Li, P., and Gu, Q. (2016) Complete genome sequence of *Lactobacillus plantarum* Lz95, a potential probiotic strain producing bacteriocins and B-group vitamin riboflavin. *J Biotechnol* 229: 1-2. Doi: 10.1016/j.jbiotec.2016.04.048.

Li, P., Gu, Q., and Zhou, Q. (2016) Complete genome sequence of *Lactobacillus plantarum* Lz206, a potential probiotic strain with antimicrobial activity against food-borne pathogenic microorganisms. *J Biotechnol* 238: 52-55. doi:10.1016/j.jbiotec.2016.09.012.

Li, P., Zhou, Q., and Gu, Q. (2016) Complete genome sequence of *Lactobacillus plantarum* Lz227, a potential probiotic strain producing B-group vitamins. *J Biotechnol 234.* doi:10.1016/j.jbiotec.2016.07.020

Li, X., Gu, Q., Lou, X., Zhang, X., Song, D., Shen, L., and Zhao, Y. (2013) Complete genome sequence of the probiotic *Lactobacillus plantarum* strain Zj316. *Genome Announc* 1,2. doi: 10.1128/genomeA.00094-13.

Liu, C. J., Wang, R., Gong, F. M.., Liu, X. F., Zheng, H. J., Luo, Y. Y., and Li, X. R. (2015) Complete genome sequences and comparative genome analysis of *Lactobacillus plantarum* strain 5-2 isolated from fermented soybean. *Genomics* 106, no. 6: 404-11. doi:10.1016/j.ygeno.2015.07.007.

Malik, S., Siezen, R. J., Renckens, B., Vaneechoutte, M., Vanderleyden, J., and Lebeer, S. (2014) Draft genome sequence of *Lactobacillus plantarum* Cmpg5300, a human vaginal isolate. *Genome Announc* 2, 6. doi:10.1128/genomeA.01149-14.

McLeod, A., Fagerlund, A., Rud, I., and Axelsson, L. (2019) Large plasmid complement resolved: complete genome sequencing of *Lactobacillus plantarum* MF1298, a candidate probiotic strain associated with unfavorable effect. *Microorganisms* 7, 8 doi:10.3390/microorganisms7080262.

Noda, M., Shiraga, M., Kumagai, T., Danshiitsoodol, N., and Sugiyama, M. (2018) Characterization of the Sn35n strain-specific exopolysaccharide encoded in the whole circular genome of a plant-derived *Lactobacillus plantarum*. *Biol Pharm Bull* 41, 4: 536-45. doi:10.1248/bpb.b17-00840.

Prechtl, R. M., Wefers, D., Jakob, F., and Vogel, R. F. (2018) Structural characterization of the surface-associated heteropolysaccharide of *Lactobacillus plantarum* Tmw 1.1478 and genetic analysis of its putative biosynthesis cluster. *Carbohydr Polym* 202: 236-45. doi:10.1016/j.carbpol.2018.08.115.

Siezen, R. J., Francke, C., Renckens, B., Boekhorst, J., Wels, M., Kleerebezem, M., and van Hijum, S. A. (2012) Complete resequencing and reannotation of the *Lactobacillus plantarum* WCSF1 genome. *J Bacteriol* 194, 1:195-6. doi: 10.1128/JB.06275-11.

Tao, X., Jiang, M., Zhang, F., Xu, F., and Wei, H. (2015) Draft genome sequence of *Lactobacillus plantarum* Wlpl04, isolated from human breast milk. *Genome Announc* 3, no. 6 (Dec 2015). doi:10.1128/genomeA.01443-15.

Tatusova, T., Ciufo, S., Fedorov, B., O'Neill, K., and Tolstoy, I. (2014) Refseq microbial genomes database: new representation and annotation strategy. *Nucleic Acids Res* 42, D553-9. doi:10.1093/nar/gkt1274.

Wang, J., Zhao, X., Tian, Z., Yang, Y., and Yang, Z. (2015) Characterization of an exopolysaccharide produced by *Lactobacillus plantarum* Yw11 isolated from Tibet kefir. *Carbohydr Polym* 125: 16-25. doi:10.1016/j.carbpol.2015.03.003.

Wang, Y., Chen, C., Ai, L., Zhou, F., Zhou, Z., Wang, L., Zhang, H., Chen, W., and Guo, B. (2011) Complete genome sequence of the probiotic *Lactobacillus plantarum* St-III. *J Bacteriol* 193, 1: 313-4. doi:10.1128/JB.01159-10.

Wang, Y., Shang, N., Qin, Y., Zhang, Y., Zhang, J., and Li, P. (2018) The complete genome sequence of *Lactobacillus plantarum* Lpl-1, a novel antibacterial probiotic producing class IIa bacteriocin. *J Biotechnol* 266: 84-88. doi:10.1016/j.jbiotec.2017.12.006.

Yang, B., Chen, H., Tian, F., Zhao, J., Gu, Z., Zhang, H., Chen, Y. Q., and Chen, W. (2015) Complete genome sequence of *Lactobacillus plantarum* Zs2058, a probiotic strain with high conjugated linoleic acid production ability. *J Biotechnol* 214: 212-3. doi:10.1016/j.jbiotec.2015.09.036.

Zhang, W., Ji, H., Zhang, D., Liu, H., Wang, S., Wang, J., and Wang, Y. (2018) Complete genome sequencing of *Lactobacillus plantarum* JDM1. *Front Physiol* 9:1689. doi:10.3389/fphys.2018.01689.
